# Paradoxical Efficacy With Rare Adverse Events: Sequential ALK Inhibitor Therapy in Lung Adenocarcinoma

**DOI:** 10.1002/ccr3.72469

**Published:** 2026-04-24

**Authors:** Kang Xie, Nie Xu

**Affiliations:** ^1^ Department of Oncology The Research Institute of Integrated TCM & Western Medicine of Chengdu University of Chinese Medicine, Chengdu Integrated TCM and Western Medicine Hospital Chengdu China

**Keywords:** ALK inhibitor, Ensartinib, lorlatinib, lung adenocarcinoma, organizing pneumonia

## Abstract

Anaplastic lymphoma kinase (ALK) inhibitors have markedly improved outcomes in ALK‐positive nonsmall‐cell lung cancer (NSCLC). While their efficacy is well documented, rare or organ‐specific toxicities remain underreported, limiting clinicians' ability to anticipate and manage adverse events during treatment or rechallenge with tyrosine kinase inhibitors (TKIs). We report the case of a 50‐year‐old woman with advanced ALK‐positive NSCLC who experienced rapid tumor regression after 1 month of treatment with ensartinib, complicated by mild bilateral interstitial pneumonitis. The pneumonitis resolved upon drug discontinuation. Following progression on second‐line chemotherapy, she was rechallenged with lorlatinib, which induced severe cutaneous toxicity that responded to corticosteroids. This is the first reported case of ensartinib‐induced interstitial pneumonitis and illustrates a dissociation between tumor response and pulmonary toxicity, as well as sequential multiorgan toxicities during ALK TKI rechallenge. These findings highlight the need for heightened vigilance, multidisciplinary management, and the development of predictive biomarkers for TKI‐associated toxicities. Future prospective studies are essential to guide safe rechallenge strategies and personalize toxicity monitoring in ALK‐positive NSCLC.

## Key Clinical Message

- Recognition of rare adverse events associated with ALK inhibitors remains limited.
- Ensartinib‐induced interstitial pneumonitis is rare and characterized by dissociation between tumor response and pulmonary toxicity.
- Multiorgan toxicity following rechallenge underscores the importance of multidisciplinary management and biomarker identification to predict susceptibility to TKI‐related adverse effects.

## 1 Introduction

Lung cancer remains the most commonly diagnosed malignancy and the leading cause of cancer‐related mortality worldwide, with approximately 85% classified as nonsmall‐cell lung cancer (NSCLC) [1]. Chromosomal rearrangements involving the anaplastic lymphoma kinase (ALK) gene occur in 3%–7% of NSCLC cases, predominantly among younger patients, never or light smokers, and those with adenocarcinoma histology [2]. Since the approval of crizotinib in 2011, nine ALK tyrosine kinase inhibitors (TKIs) have been introduced as standard therapies for ALK‐positive NSCLC. New‐generation ALK TKIs, including alectinib, brigatinib, ensartinib, and lorlatinib, have demonstrated superior efficacy compared with crizotinib [3, 4, 5]. Ensartinib is the first ALK inhibitor developed independently in China. A recent meta‐analysis suggests that ensartinib may represent the most effective first‐line treatment for ALK‐positive NSCLC in Asian populations, with a median progression‐free survival (PFS) of 25.8 versus 12.7 months for crizotinib [6].

Despite their clinical benefit, ALK TKIs can cause rare but potentially life‐threatening adverse events (AEs), including drug‐induced interstitial pneumonitis. While most AEs are Grade 1 or 2 and primarily dermatologic, ensartinib‐specific toxicities commonly include rash (67.8%), elevations in aspartate aminotransferase (37.8%) and alanine aminotransferase (48.3%), pruritus (26.6%), nausea (22.4%), constipation (20.3%), edema (21.0%), and anemia (14.0%) [7]. Interstitial pneumonitis associated with ensartinib has not been previously documented in real‐world settings. Here, we report a case of advanced lung adenocarcinoma that responded rapidly to first‐line ensartinib but developed grade 1 interstitial pneumonitis. Following disease progression on chemotherapy, lorlatinib rechallenge led to severe cutaneous toxicity. This case underscores the clinical dilemma posed by rare but serious toxicities that complicate the therapeutic management of ALK‐positive NSCLC.

## 2 Case History‐Examination

**FIGURE 1:**
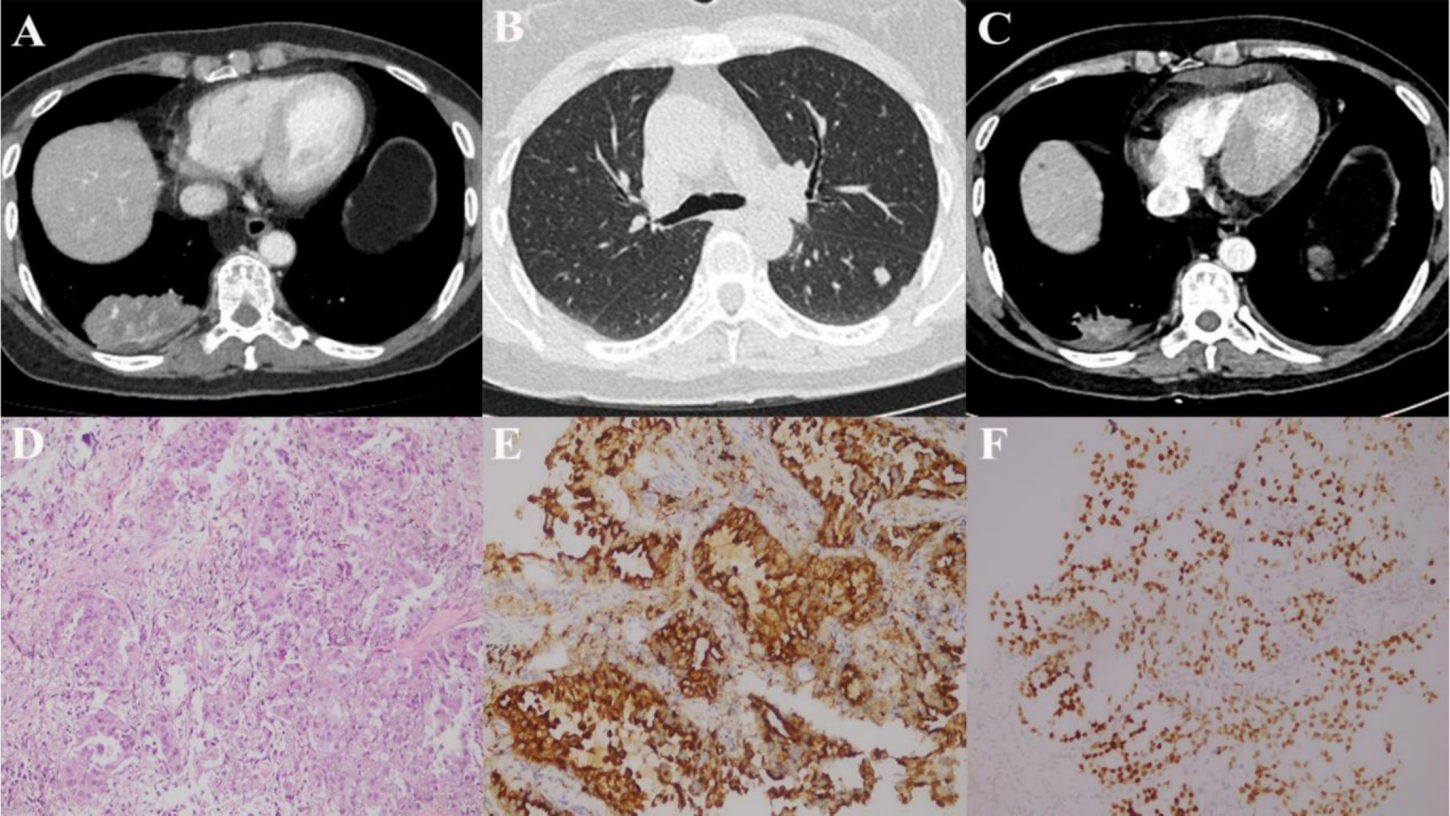
(A) The first chest CT scan (A 6.7 × 4.5 cm soft tissue mass with burr development in the lower lobe of the right lung). (B) Metastatic nodule in the right lower lobe of the lung. (C) After 1 month of oral ensartinib administration, the primary lesion showed significant shrinkage. (D) (HE×200) The histopathological investigation revealed adenocarcinoma. (E) (IHC × 200) NapsinA exhibits diffuse moderate to strong positive expression in tumor cells. (F) (IHC × 200) TTF‐1 shows mild to moderate positive expression in cells. CT, computed tomography.

A 50‐year‐old woman presented with chest tightness. Contrast‐enhanced chest computed tomography (CT) revealed a lobulated, spiculated mass in the lower lobe of the right lung measuring 6.7 × 4.5 cm, with heterogeneous enhancement. Multiple bilateral pulmonary nodules and enlarged mediastinal lymph nodes were also identified (Figure 1A,B). Abdominal CT, cranial magnetic resonance imaging (MRI), and bone scintigraphy showed no evidence of metastasis. To confirm the diagnosis, a percutaneous transthoracic lung biopsy was performed. Histopathological evaluation revealed lung adenocarcinoma. Immunohistochemical staining showed CK(+), CK5/6(−), P40(−), CK7(+), TTF‐1(+), Napsin A(+), CK20(−), Villin(+), CEA(+), Syn(−), CgA(−), SMARCA4 (retained), P504S(−), and Ki‐67 30% (Figure 1D–F).

## 3 Differential Diagnosis, Investigations, and Treatment

**FIGURE 2:**
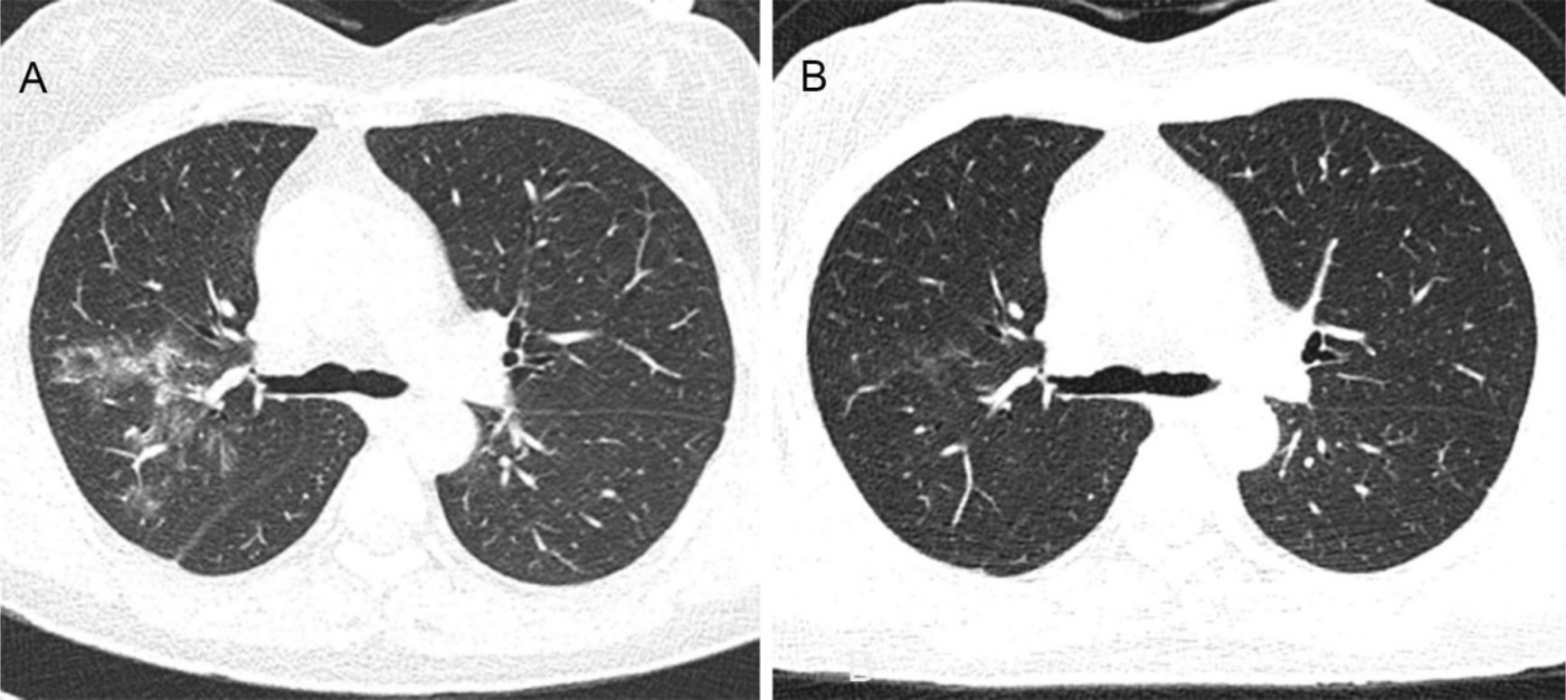
(A) Chest CT showing multiple patchy lesions appeared in the bilateral lungs, especially in the right upper lung. (B) Patchy shadows in the right lung dissipated after 1 week of treatment.

The tumor was classified as cT4N2M1c, stage IVB, according to the 8th edition of the AJCC (American Joint Committee on Cancer) staging criteria. Next‐generation sequencing revealed an EML4‐ALK gene fusion with a variant abundance of 7.69%. The patient was initiated on oral ensartinib 225 mg once daily. After 20 days of treatment, she developed a Grade 2 maculopapular rash with pruritus, along with a mild cough and chest tightness. Vital signs, including oxygen saturation and temperature, remained normal. Repeat contrast‐enhanced chest CT demonstrated a marked reduction in the right lower lobe mass (3.5 × 1.8 cm) (Figure 1C). However, new bilateral upper lobe patchy opacities were noted. Infectious etiologies, including bacterial and viral pneumonitis, were excluded. Interstitial pneumonia secondary to ALK TKI therapy was suspected. Ensartinib was discontinued, and the patient received oral dexamethasone 5 mg once daily for 1 week, leading to complete radiologic resolution of pulmonary infiltrates (Figure 2).

To reduce the risk of recurrent interstitial pneumonitis, ensartinib was not reinitiated. The patient began systemic chemotherapy with pemetrexed disodium (500 mg/m^2^) plus cisplatin (75 mg/m^2^) and bevacizumab (15 mg/kg) every 3 weeks. After 6 cycles, the primary tumor showed progression. Given the initial favorable response to ensartinib and the limited toxicity (Grade 1 interstitial pneumonitis and mild rash), a rechallenge with ALK TKI was considered. Following detailed counseling, the patient and family consented to treatment with lorlatinib 100 mg orally once daily, under the supervision of her son.

## 4 Outcome and Follow‐Up

After 8 days of lorlatinib, the patient developed a diffuse, bright red maculopapular rash with severe pruritus, facial and peripheral edema, and a fever of 39°C. Laboratory studies revealed no eosinophilia and normal hepatic and renal function. A Grade 4 cutaneous adverse reaction was diagnosed, and lorlatinib was discontinued. Importantly, chest CT showed no evidence of interstitial pneumonitis, and further tumor regression was noted. The patient was treated with methylprednisolone, oral prednisone, calcium gluconate, and cephalosporin antibiotics. The rash resolved completely within 2 weeks. She was discharged in stable condition with plans for continued outpatient follow‐up.

## 5 Discussion

In patients with lung adenocarcinoma treated with ALK TKIs, interstitial pneumonia can be initially misdiagnosed as infectious pneumonitis or disease progression.

Interstitial pneumonia is a rare but potentially fatal AE associated with ALKTKI therapy. A comprehensive understanding of this drug‐related pulmonary toxicity is of significant importance in clinical practice. A meta‐analysis incorporating 26 clinical trials with a total of 4752 patients demonstrated that the overall incidence of all‐grade pneumonitis was 2.92% (95% confidence interval [CI]: 1.79%–4.27%), while the incidence of high‐grade (Grade 3–4) and fatal (Grade 5) pneumonitis was 1.42% (95% CI: 0.84%–2.12%) and 0.09% (95% CI: 0.00%–0.28%), respectively [8]. Another analysis based on the US Food and Drug Administration Adverse Event Reporting System (FAERS) revealed that among 20,064 ALK‐TKI‐related AEs reports, interstitial lung disease (ILD)‐related reports accounted for 3.2% (640 cases) [9]. A network meta‐analysis comparing the risk differences among various ALK‐TKIs indicated that brigatinib was associated with the highest risk of pneumonitis [10].

Regarding the timing of onset, analysis of the FAERS database demonstrated that the median time to onset of ALK‐TKI‐related ILD was 53 days (first quartile: 12 days; third quartile: 209 days), with over 70% of cases occurring within the first 2 months of treatment.

Multiple studies consistently indicate that the incidence of pneumonitis is significantly higher in Japanese populations compared with non‐Japanese populations. The incidence of all‐grade pneumonitis was 6.25% in Japanese cohorts versus 1.14% in non‐Japanese cohorts (*p* < 0.001). Multivariate meta‐regression analysis confirmed that the odds ratio (OR) for pneumonitis in Japanese populations was 4.329 [11]. A study on alectinib combined with thoracic radiotherapy showed that increasing age was an independent risk factor for treatment‐related pneumonitis (OR = 1.103) [12].

The incidence of pneumonitis in patients receiving ALK‐TKIs after prior chemotherapy was significantly higher than in those receiving them as first‐line therapy (all grades: 7.73% vs. 2.26%; high grade: 3.64% vs. 1.26%) [8]. Concomitant use of proton pump inhibitors (PPIs), amlodipine, and magnesium oxide significantly increased the risk of ILD (*p* < 0.05) [9]. Alectinib combined with thoracic radiotherapy significantly increased the risk of treatment‐related pneumonitis, with an incidence of 62.9% in the combination therapy group and grade ≥ 2 pneumonitis accounting for 35.5% [12]. Risk factors included the duration of alectinib use and the lung V30 dose parameter.

According to the prescribing information for ALK‐TKIs such as ensartinib, alectinib, and lorlatinib, the drug should be permanently discontinued in cases of treatment‐related ILD of any grade. Standard management includes immediate drug discontinuation and exclusion of other etiologies, such as infection or tumor progression. Glucocorticoid therapy and supportive treatment should be administered based on the severity of the condition.

Whether to switch to another ALK‐TKI after developing ILD remains controversial. Some argue that ILD may represent a class effect of ALK‐TKIs and that switching to another TKI could lead to recurrence of pneumonitis. However, case reports have described successful switching to alternative TKIs. Myall et al. reported two cases of successful lorlatinib use after alectinib‐induced pneumonitis [13]. Currently, there is a lack of prospective data to guide decision‐making, and no reliable biomarkers exist to predict which patients can safely tolerate a second TKI.

To our knowledge, this is the first real‐world report of acute interstitial pneumonitis associated with ensartinib. Follow‐up CT after 1 month of therapy revealed bilateral scattered opacities suggestive of interstitial inflammation, while the primary tumor had significantly regressed. Infectious etiologies were excluded. Based on CTCAE version 5.0 (Common Terminology Criteria version 5.0) criteria [14], the patient was diagnosed with grade 1 interstitial pneumonitis, which resolved following ALK TKI discontinuation and corticosteroid therapy. In accordance with guideline recommendations, the patient was transitioned to systemic chemotherapy to prevent recurrence of severe pulmonary toxicity. However, following 6 cycles, disease progression was observed. Given the lack of effective alternatives, the possibility of ALK TKI rechallenge was discussed with the patient and her family. Lorlatinib was selected for its relatively favorable pulmonary toxicity profile. Unfortunately, after 8 days of treatment, the patient developed a severe Grade 4 cutaneous reaction without recurrence of pneumonitis, necessitating drug discontinuation. Despite the short duration of ALK TKI therapy, the patient achieved notable tumor reduction.

This case illustrates the clinical challenges posed by sequential, rare, and serious TKI‐related toxicities. The occurrence of interstitial and cutaneous hypersensitivity reactions in close temporal sequence suggests the possibility of a shared immunologic pathway involving ALK TKIs such as ensartinib and lorlatinib. Interstitial pneumonia associated with ALK inhibitors typically responds well to corticosteroids in approximately 30% of patients upon TKI reintroduction or discontinuation. In rare cases, subclinical interstitial pneumonia may persist without symptomatic relapse. As the use of ALK TKIs continues to expand, early recognition and intervention for pulmonary toxic treatment, with symptomatic relief occurring within days to weeks. Recurrence has been reported—particularly in sensitized individuals—are critical to reducing morbidity and mortality. Interstitial pneumonitis should be considered in patients with new bilateral lung infiltrates in the absence of identifiable infectious or neoplastic causes, and prompt cessation of therapy with initiation of corticosteroids remains the cornerstone of management.

## 6 Conclusions

We report a rare case of interstitial pneumonitis in a patient with advanced ALK‐positive lung adenocarcinoma treated with ensartinib, a toxicity not previously documented in the literature. The observed dissociation between tumor response and pulmonary toxicity, along with sequential organ‐specific adverse effects following ALK TKI rechallenge, suggests a potential immune‐mediated cross‐reactivity between different ALK inhibitors. This case expands the current understanding of ensartinib‐associated toxicity and underscores the importance of genomic profiling and multidisciplinary management in ALK‐positive NSCLC. Prospective studies are warranted to identify predictive biomarkers of TKI‐related toxicities and to establish safe rechallenge and treatment strategies.

## Author Contributions

**Kang Xie:** conceptualization, data curation, formal analysis, investigation, writing – original draft. **Nie Xu:** conceptualization, data curation, formal analysis, investigation, project administration, writing – review and editing.

## Funding

This work was supported by the Research Project of Traditional Chinese Medicine in Sichuan Provincial Administration of Traditional Chinese Medicine (Grant [2025]6‐199); Chengdu Medical Research Project (Grant 2023384); Chengdu Municipal Health Commission, Chengdu University of Traditional Chinese Medicine Joint Innovation Fund (Grant [2024]33‐36).

## Consent

Written informed consent was obtained from the individual(s) for the publication of any potentially identifiable images or data included in this article.

## Conflicts of Interest

The authors declare no conflicts of interest.

## Data Availability

The data presented in this study are available in this article.
